# Ant-App-DB: a smart solution for monitoring arthropods activities, experimental data management and solar calculations without GPS in behavioral field studies

**DOI:** 10.12688/f1000research.5931.3

**Published:** 2015-05-12

**Authors:** Zeeshan Ahmed, Saman Zeeshan, Pauline Fleischmann, Wolfgang Rössler, Thomas Dandekar

**Affiliations:** 1 Department of Quantitative Health Sciences, University of Massachusetts Medical School, Greater Boston, MA, 01605, USA; 2 Department of Neurobiology and Genetics, Biocenter, University of Wuerzburg, Wuerzburg, 97074, Germany; 3 Department of Bioinformatics, Biocenter, University of Wuerzburg, Wuerzburg, 97074, Germany; 4 Department of Behavioural Physiology and Sociobiology, Biocenter, University of Wuerzburg, Wuerzburg, 97074, Germany; 5 EMBL, Structural and Computational Biology Unit, Heidelberg, 69117, Germany

**Keywords:** Arthropod behaviour, behavioural ecology, Data collection, GPS/GIS, Android, Tracking

## Abstract

Field studies on arthropod ecology and behaviour require simple and robust monitoring tools, preferably with direct access to an integrated database. We have developed and here present a database tool allowing smart-phone based monitoring of arthropods. This smart phone application provides an easy solution to collect, manage and process the data in the field which has been a very difficult task for field biologists using traditional methods. To monitor our example species, the desert ant *Cataglyphis fortis*, we considered behavior, nest search runs, feeding habits and path segmentations including detailed information on solar position and azimuth calculation, ant orientation and time of day. For this we established a user friendly database system integrating the Ant-App-DB with a smart phone and tablet application, combining experimental data manipulation with data management and providing solar position and timing estimations without any GPS or GIS system. Moreover, the new desktop application Dataplus allows efficient data extraction and conversion from smart phone application to personal computers, for further ecological data analysis and sharing. All features, software code and database as well as Dataplus application are made available completely free of charge and sufficiently generic to be easily adapted to other field monitoring studies on arthropods or other migratory organisms. The software applications Ant-App-DB and Dataplus described here are developed using the Android SDK, Java, XML, C# and SQLite Database.

## Introduction

The traditional way of collecting and managing the data in behavioral field studies has been a tedious and laborious task. It requires the marking and monitoring of arthropods in the field along with the manual entry and management of the data about marked insects, feeders and experiments. Moreover, it becomes extremely complex, when, for example, behavioral biologists and ecologists have to estimate solar position and time without any GPS system or internet access in remote and wild regions. In field studies monitoring of arthropods requires an easy to handle application, monitoring movement as well as behavioral parameters. A desktop application installed in a laptop may not be a reliable solution, due to humid, warm and uncertain weather conditions. For this, coupling modern database technology with a low weight or low cost smart phone application can provide a strong, user-friendly tool to adopt ^1^.

Several beneficial applications have already been developed to improve the field of ecology e.g. animal and plant georeference phenological recording ^2^, crowd-sourcing ^3, 4^, gearing community developmental research ^5^ with scientific approach ^6^, collecting data in the field with a GPS system ^7^ or a GIS system ^8^. Despite some existing useful technological solutions in the field, we found some gaps that still need to be addressed. For instance, there is no specific smart phone or tablet application available for optimized arthropod monitoring in the field without internet connection or online GPS to access solar position and time at a specific location. Effective small animal monitoring requires an application with an efficient data management system and the ability to estimate solar position and time without a GPS or GIS system.

We offer a thoroughly developed generic solution which can easily be adapted to investigate behavioral parameters in small animals such as insects and is hence made freely available for such efforts. The present application was originally developed and optimized for monitoring a desert ant species, *Cataglyphis fortis*, a social insect, which mainly uses a polarized-skylight based sun compass for path integration to be able to orient and home in featureless environments ^9– 11^. In addition, olfactory cues are used for orientation close to the nest entrance ^12^.

In areas inhabited by *Cataglyphis fortis* (salt flats in North African deserts)–the lack of prominent visual landmarks means that ants mostly rely on celestial cues. High temperatures and an unpredictable distribution of food force the ants to make long-winded search runs to then return in a straight path back to the nest ^13^. The high level of complexity in orientation and extreme environmental conditions requires novel tools to monitor the ant’s behavior in an easy to use fashion that allows production of accurate data: for example, the direction of foraging and homing runs with relation to solar time, solar azimuth or time of the day. This information can be combined to register individually color-code marked ants (using a three dot color code) at different times and locations during the experiment.

A large amount of computational research has been performed with regard to behavior studies, for instance in artificial intelligence ^14^ and different approaches have been proposed e.g. 15– 27. However, the specific field experimental paradigm related to polarized skylight compass orientation leads to different, specific kinds of information where an optimal, easy-to-handle tool and database has not yet been established. Without any swift and effectual technological solution, the experimentation process may become very complex and time consuming, as the observer has to do many tasks at one time e.g. managing information about the running experiment, food at feeders, color marking of the ants, separating registered and unregistered ants, and observing the continuous change in the current time of the location, solar time, solar zenith and azimuth angle.

To cope with this, we propose a new product line architecture (PLA) based scientific solution, the Ant-App-DB; a user friendly, smart phone and tablet application, helpful in efficient management of experimental data including location, date, time, geographical measurements, feeders, registered and unregistered ants ^28^. We also present another new multi document interface (MDI) desktop application Dataplus; that enables quick data transfer from the smart phones exported database file and conversion into the Microsoft excel format for data storage and further data analysis.

The major reason for developing the smart phone application is to have a low weight, user friendly way of managing experimental processes along with data sharing. Moreover, it is also worthy to take advantage of the advanced mobile computing and service provision of this era, which offers small sized devices (easy to carry, usable worldwide and affordable), embedded with extra durable (rechargeable and replaceable) batteries, internal and external memory cards and most of all temperature resistance with the ability to withstand extreme conditions such as those found in deserts where laptops or other computational devices can experience problems.

The following sections of the manuscript explain the methodology, architected software and database designs, and implementation with modular description of the application.

## Methods

Ant-App-DB is a well-developed application, following the principles of three layered Butterfly ^29, 30^ software development model towards scientific software engineering (SSE), integrating formal Unified Modelling Language (UML) ^31, 32^ perspectives and incorporating Human Computer Interaction (HCI) design patterns.

The overall software engineering process of the Ant-App-DB is well planned, as initially the possible number of requirements were gathered and discussed, abstract application designs were architected, and mockup designs of graphical user interface (GUI) were constructed following a brain storming session by the authors and other colleagues. Implementable designs (use case, database, dataflow, work flow, system sequence, class and components) were then drawn based on the finalized functional requirements, the most suitable technologies (both software and hardware) were chosen, comprehensive prototype development was performed and the end product was successfully deployed and tested in-house.

**Figure 1.**
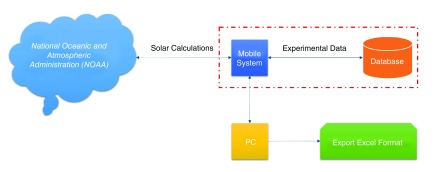
Ant-App-DB Conceptual Architecture. This figure shows the conceptual architecture of the Ant-App-DB application, which consists of five main components: mobile system, database, solar estimations using recommended algorithms by the National Oceanic and Atmospheric Administration (NOAA), personal computer and exporting data in Microsoft Excel format.

The conceptual architecture of Ant-App-DB ( Figure 1) is divided into five different modules: Mobile System, Database, NOAA, Personal Computer and Export Excel Format. ‘Mobile system’ is the smart phone application to be used in the experiments on the field, ‘Database’ is the embedded data management system in the smart phone, ‘NOAA’ is an integrated module in smart phones to estimate solar timing and angles using different astronomical algorithms recommended by the National Oceanic and Atmospheric Administration.

The personal computer module uses the desktop application (Dataplus) to extract data from the smart phone database and then converts into the Microsoft Excel format.

## Implementation

The designed and implemented methodology is explained in the following UML notation and semantics for use case, activity, dataflow, system sequence, class, component and database (entity relationship) diagrams (Please see details in the Supplementary material).

**Figure 2.**
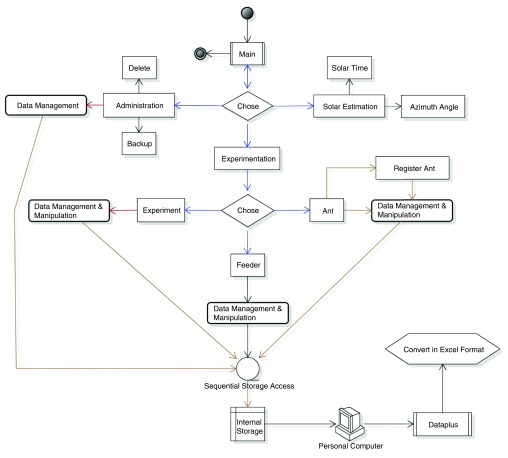
Workflow of Ant-App-DB. This figure shows the application’s activity workflow, which starts with the Main interface with four categorized options: Close, Administration, Solar Estimation and Experimentation. Choosing Close (default return option in almost all Android based smart phones), user can exit from the application. Using Administration an authorized user can delete or backup the data and via Solar Estimation user can calculate different solar timings and angles. Experimentation option allows users to enter and manage the information related to the Experiments, Feeders and Ants. Data from all applications are stored in the internal storage with sequential access, which then can be exported to the personal computer and, using Dataplus, can be converted in Microsoft Excel format.

The activity work flow ( Figure 2) starts with the main GUI of the application (describing the available options provided to the observers), which is further categorized as three different processes: administration, experimentation, and solar estimation. Administration offers a secured access to the authorized users/observers for deleting or creating backups of the existing records in the internal or external storage locations, which then can be exported, reused and shared. Experimentation allows users to manipulate and manage information related to experiments, feeders (optional), registration of ants and ants to be used during experiments. Moreover, it also offers an additional interface (Quick Ant) to fasten the experimentation process and presents stored results in tabular form. The solar estimation process allows users to approximate the solar time and azimuth angle using any given (valid) date, time, UTC time zone, longitude and latitude. Finally, the data (SQLite database) can be exported from the smart phone application to a personal computer and then using Dataplus can convert data into the Microsoft Excel sheet format.

**Figure 3.**
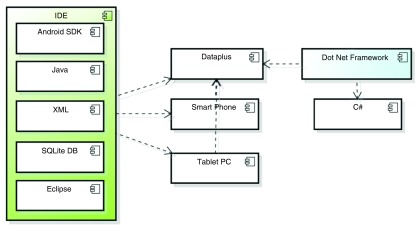
Components of Ant-App-DB. This figure shows wiring of the components of the Ant-App-DB. It consists of four major components: IDE (with five sub-components: Android SDK, Java, XML, SQLite DB, Eclipse), Personal Computer (PC), Smart Phone, Tablet PC and Microsoft Dot Net Framework (including C# programming language).

As shown in the component diagram ( Figure 3), Ant-App-DB is an Android operating system based application (tested using a Sony Xperia Z1 smart phone and the Android SDK based emulator). Eclipse Integrated Development Environment (IDE) was used for the entire smart phone application development using Java programming language, XML, Android SDK and SQLite database for embedded database scripting. The Dataplus module was developed in C-Sharp programming language in Microsoft Dot NET Framework.

Following the designed sequence of the application, the implemented source code is divided into two sections: GUI and the logic of the program. The designed GUI (8 horizontal and 8 vertical pages) are implemented in XML and the main logic of the application is implemented in Java programming language.

***Data management.*** To manage the application’s data, we designed a normalized entity relationship model and implemented this in SQLite database management (please see Supplementary material for details).

**Figure 4.**
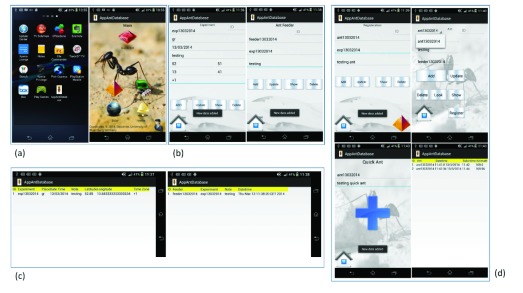
Graphical User Interface (GUI) presentation of the Ant-App-DB. Figure 4(a) is the Android based smart phone’s graphical user interface (Sony Xperia Z1). It also presents the main graphical user interface of the application with 6 important buttons leading to 6 different interfaces. The green computer button navigates to the Experiment interface, the yellow bell button directs to the Feeder interface, the orange pyramid button routes to the Ant interface, the red twisted button leads to the Quick Ant interface, the blue earth button proceeds to the Approximate Solar Calculations, and the button with a man in a suit image goes to the Admin interface. Figure 4(b) is the Experiment and Feeder interface, where experimental and feeder-related information is entered, managed, deleted and viewed. Figure 4(c) shows an example for a successful input of experimental and feeder data in the database. Figure 4(d) presents the Registration, Ant, and Quick Ant graphical interfaces, where unregistered Ants can be registered, and, for example, their feeder-visit related information can be managed into the system. Moreover, it also successfully transfers Ant data in the database.

The Ant-App-DB is divided into six major interlinked GUIs: Main, Experiments, Ant Feeder, Registration, Ant, and Quick Ant. The main GUI of the application can be accessed via a white image (an ant on a white background) marked by a red line ( Figure 4a). It has six important options leading to six different GUIs. The green computer button navigates to the Experiment interface, the yellow bell button directs users to the Feeder interface, the orange pyramid button routes to the Ant interface, the red twisted button provides a connection to the Quick Ant interface, the blue earth button proceeds to the Approximate Solar Calculations and the button with the image of a man in a suit is linked to the Admin interface.

The Experiment interface ( Figure 4b) is the first and the most important module of the application, where experiment-related information needs to be entered and managed. This module asks the user to provide information about the name of the experiment, the date and time of the experimentation, and any additional notes. Furthermore, it asks the user to provide geographical information about the location of the experimentation, which includes the latitude, longitude and UTC time zone. It allows the user to give positioning information in degrees and/or minutes. The user can update existing information by editing or deleting information with reference to the automatically generated ID, and can view the stored information in tabular form.

The Ant Feeder interface ( Figure 4b) manages information about the used feeders during experimentation which is important, though optional. In the GUI ‘Experiment’, the user can update existing information in Feeder by editing, deleting information with reference to the automatically generated ID, and viewing the stored information in tabular form. The stored data using Experiment and Feeder GUIs is presented in Figure 4(c).

The Registration interface ( Figure 4d) is another very important module of the application. It can only be accessed from the Ant interface and is used to register the ants before experimentation. It asks the user to give information (names, numbers) about used (color marked) ants and to select the experiment (from the list of the experiments). Furthermore, the user can update existing information by editing, deleting with reference to the automatically generated ID, and viewing the stored information in tabular form.

The Ant interface is divided into two modules: Ant and Quick Ant ( Figure 4d). The major difference is the availability of the options, as the Ant interface allows the user to select registered ants at different feeders. It also provides options to perform data manipulation. However Quick Ant allows the user to only select the name of the Ant from the registered ants list. Both interfaces have in common the provision of an additional notes field and the automatic extraction of the information (from the database) about associated experiments. The Note option helps the user to save any additional experimental information helpful for geographical details (latitude, longitude and time zone), or to calculate, save and manage the solar time and azimuth angle. The main reason for dividing the Ant interface into two different modules is to help speed up the experimental process. Examples for stored Ant and Quick Ant results are shown in Figure 4(d).

***Solar estimations.*** The solar estimation is a very important section of this application, as it increases the practical value of experiments in extreme conditions, especially since there is no internet available in deserts, and the use of printed tables is time consuming. Therefore, it is nearly impossible to compute solar time and azimuth angles manually (by use of tables) in the field, for example to precisely register each visit of a color-marked ant to a feeder. These calculations normally have to be done afterwards in time consuming sessions.

The solar estimation module works independently as well as in combination with the experimental data management system. Using different astronomical algorithms ^33– 35^, it estimates approximate Gregorian Day Number, Decimal Day, Decimal Day of the Year, Fractional Year, Equation of the Time, Declination, Solar Time Offset, Solar Time Solar Zenith Angle, Solar Hour Angle, Solar Azimuth Angle and Solar Noon. For any registered ant, for example its visits to a feeder or other observed locations, it will automatically extract the information about latitude and longitude from the associated experiment. Current date and time is entered automatically and saved with the information about solar timing and angles.

The user provides information about latitude and longitude, and adds manual or automatic date and time information. The current Gregorian calendar day number is calculated:

*Day of the year* = 365 * *year* + *year*/4 – *year*/100 + *year*/400 + (( *month*+1) * 306)/10 + ( *day* – 62)

Next, fractions of a full day are considered:

***Decimal day* = ( *dhour*/24) + ( *dminutes*/1440)**

Both are combined for the decimal day of the year:

***Decimal day of the year* = *Day of the year* + *Decimal day***

The Fractional Year uses PI (3.14) and hour (current time in hours):

*Fractional Year* = (2 * *PI*/365) * ( *Decimal day of the year* – 1 + (( *hour* – 12)/24))

Now, the equation of time and declination are calculated:

*Equation of Time* = 229.18 * (0.000075 + 0.001868 * *cos* ( *Fractional Year*) – 0.032077 * *sin* ( *Fractional Year*) – 0.014615 * *cos* (2 * *Fractional Year*) – 0.040849 * *sin* (2 * *Fractional Year*))

*Declination* = 0.006918 – 0.399912 * *cos* ( *Fractional Year*) + 0.070257 * *sin* ( *Fractional Year*) – 0.006758 * *cos* (2 * *Fractional Year*) + 0.000907 * *sin* (2 * *Fractional Year*) – 0.002697 * *cos* (3 * *Fractional Year*) + 0.00148 * *sin* (3 * *Fractional Year*)

Solar time offset and solar time are estimated:

*Solar Time offset* = 4 * ( *longitude* – (15 * *Time zone*)) + *Equation of Time*

*Solar Time* = *hour* * 60 + *min* + *sec*/60 + *Solar Time Offset*

Using solar zenith angle and solar hour angle (ha), the azimuth angle is estimated:

*Solar Zenith Angle* = ( *sin* ( *Latitude*) * *sin* ( *Declination*)) + ( *cos* ( *Latitude* * *cos* ( *Declination*) * *cos* ( *ha*))

*Solar Hour Angle* = *Solar Time* * 60

*Azimuth Angle* = *atan*2 ( *sin* ( *ha*)), *cos* ( *ha*) * sin (lat) – *tan* ( *Declination*) * *cos* ( *Latitude*))

Finally, the solar noon is calculated:

*Solar noon* = 720 + (4 * *Longitude*) – *Equation of Time*

**Figure 5.**
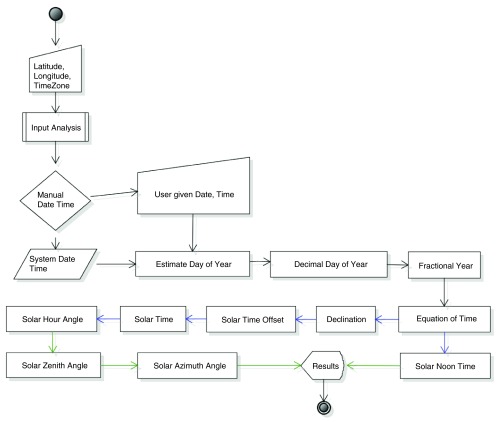
Sequential Process of Solar Calculations. This Figure presents a sequential process for the solar calculations, where output from almost each process directly or indirectly is used as the input for the calculations of the following process.

The workflow of the solar estimation module starts with the user entered information about the latitude and longitude with manual or automatic (from the system) date and time information. First, the day of the year is estimated, then decimal day of the year, then Fractional Year, then Equation of Time, then Solar Noon Time, then Declination, then Solar Time Offset, then Solar Time, then Solar Hour Angle, then Solar Zenith and Solar Azimuth Angle. At the end all results are presented in textual format ( Figure 5).

The obtained results match well with the results produced by solar calculators provided by or linked with the National Oceanic & Atmospheric Administration (NOAA) (99.99% accurate Solar Time and 99.8% accurate Azimuth Angle, please see attached supplement material for more details).

The online NOAA solar calculation, however, is not always accessible (as, for example, it is the case in desert ant observations without internet access), and it is time-consuming to implement (not all necessary steps are readily apparent from the NOAA web site), or needs to be consulted after the experimental procedure. Our main aim was to have an easy-to-use, stand-alone application to register individual ants together with positional and behavioral data, and to be able to immediately import all calculations and observations in a custom-made database.

***Data administration.*** The administration module of the application provides two major options: clearing or deleting records, and creating data backups. Only authorized users can delete the records of the Ant, Registration, Feeder and Experiment interfaces (individually or all at once) by entering a security key into the system. The generated backup of the data is stored in the external (e.g. SD card) or internal storage location of the smart phone or tablet, which can be later copied, exported and reused. The exported file’s name is based on the following structure: Ant-App-DB, current date and time in the mobile system. This helps in preventing duplication and/or replacement of data that has already been backed up (please see Supplementary material for more details). The exported data file can be shared using common Android smart phone features including internet services, USB connection, Bluetooth etc.

***Dataplus.*** Dataplus is another important module of the application which helps observers in transferring the data from the smart phone application generated SQLite database file into Microsoft Excel format for future use, analyses, sharing and backup using a personal computer. Moreover, it is possible to combine multiple exported data files from different series of experiments.

**Figure 6.**
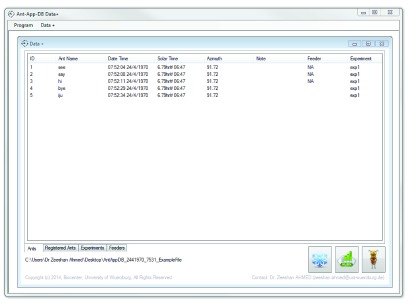
Graphical User Interface (GUI) presentation of the Dataplus. Figure 3 shows that the data is exported from Ant-App-DB in SQLite database file, which is loaded into the Dataplus module by clicking the small Ant icon button. It then can be converted into Microsoft Excel format by pressing the Excel icon button. The button with the Snowflake icon is used to remove the data.

Dataplus ( Figure 6) is a desktop MDI application, designed and developed following the concepts of the Butterfly Model ^29^ in C-sharp programming language. The application is very simple to use and install, but can only be configured using a Microsoft Windows platform.

## Operation

Ant-App-DB is very simple to use and install, but can only be configured on Android based smart phones and tablets, while Dataplus is a desktop application that can only be configured on a Microsoft Windows platform (preferred OS version: 7). Therefore, the installation of Ant-App-DB is a two-step process. The application can be configured and installed on smart phones and personal computers following the instructions in the Supplementary material.

**Figure 7.**
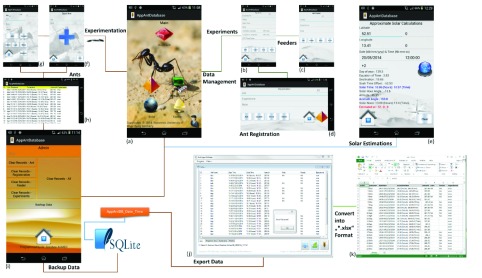
Ant-App-DB and Dataplus operational work flow. This figure presents the real time work flow of the different modules of the application. It shows the main menu ( Figure 7a), experiment ( Figure 7b), feeder ( Figure 7c) and registration of ants interfaces ( Figure 7d), approximate solar calculations ( Figure 7e), Quick Ant ( Figure 7f), Ant ( Figure 7g, Figure 7h), Admin ( Figure 7i), Dataplus interfaces ( Figure 7j), and the Microsoft Excel file format “.xlsx” ( Figure 7k).

An example operational workflow of Ant-App-DB and Dataplus is presented and briefly explained in Figure 7. As shown in Figure 7a, the observer is required to first run the application and access the different modules of the application using the main GUI.

The most important steps are to provide details about the experiment ( Figure 7b), registered color-marked ants ( Figure 7c) and feeders ( Figure 7d). Before starting the experiment, the observer can also estimate the solar position and time using the Approximate Solar Calculation module ( Figure 7f).

Later, during the experimental process, the observer is only required to run the module Quick Ant ( Figure 7f), select each color-marked and registered ant at its visit, and press the button ‘Plus’ sign. The module Ant ( Figure 7g) also offers a similar option to Quick Ant but it is recommended to use Quick Ant to avoid any unnecessary clicks etc. The results are stored in the created database, which the observer can view (e.g. Figure 7h).

The results data can be deleted or backed up using the module Admin ( S-Figure 7i), which can be copied to the personal computer and converted in to the Microsoft Excel format ( Figure 7k) using Dataplus ( Figure 7j).

## Discussion

We have tested and validated the Ant-App-DB application by successfully executing and performing available tasks, e.g. entering and storing data using the Experiment, Feeder and Registration Interface modules. We have also tested and validated the deletion and back up of data using the Admin module, as well as different solar estimations using different input values (date, time, latitude, longitude and time zone). Moreover, we have compared and confirmed the estimated solar results with NOAA.

The App has the capability to use multiple smart phones and to synchronize data between users using common smart phone features together with our Dataplus desktop application. A real time backup is another important feature. It is possible to extract, share and combine the experimental data generated during one or multiple experiments by one of multiple users using different smart phones. The data can then be exported into Microsoft Excel format for further editing and analysis.

In the future, this application can also be enhanced by adding more computation and data management features to assist the observers during the experiments, e.g. by linking it to service (if available in the required precision) of GPS to get highly accurate geographical positions, sharing data using internet services (if available), and searching data using natural language based queries. Based on the user’s feedback we can also improve the GUI and other features.

Several features of Ant-App-DB are favorable compared with other available solutions (e.g. Etholog ^36^, JWatcher ^37^, Nolduss EthoVison ^38^, Cybertracker & Animal Behavior ^39^) for effective and efficient insect monitoring such as shown by 2– 8. The most significant advantages are that it does not necessarily require any GPS and/or GIS systems, that it can be used on any Android based device without internet service, that it allows Subscriber Identity Module (SIM) card and external SD card to be used to manage the experimental data, and that it enables estimations of solar position and time. Additionally, unlike other applications, it provides a desktop application which helps extracting the data from the smart phone’s database and to convert it to Microsoft Excel formats for further analyses and data sharing. Moreover, Ant-App-DB is highly user friendly compared to other applications, as it offers ‘One Click’ operation during the experimental procedures in the field. Only few steps are needed to adapt the software for use in behavioral experiments with other arthropods or other animal species. To perform more extensive changes to the configuration, a software engineer is needed. For instance, to monitor flight time and tracks, or locomotory paths, the application needs the integration of a suitable 2D or 3D tracking module. This, potentially, could be integrated into the modular system (see Supplementary material). Overall, the system is configured in a way that other observational modules or modes can be integrated once they are properly programmed and tested.

## Conclusions

Ant-App-DB couples a database and database conversion tool with direct access and data input using a smart phone application. We have used the application in the field and have found it to be a user friendly database tool developed for behavioral research on *Cataglyphis fortis*, in particular managing experimental data and calculating observation data such as solar timing and position monitoring. However, all features, software code and database as well as Dataplus application are sufficiently generic to be easily adapted to other field monitoring studies on other arthropods (e.g. on honey bees, fruit fly etc.) or, for example, other migratory animal species. The Ant-App-DB is available to interested non-commercial users free of charge.

## Software availability

### Software access

The software executables are freely available at the following web link: http://www.neurogenetics.biozentrum.uni-wuerzburg.de/en/project/services/ant_app_db/

The software download section provides three files in total: Ant-App-DB’s APK file to be installed in the Android based smart phones, Dataplus’s executable setup to be installed on the Microsoft Windows platform and an example dataset (SQLite database file, generated by the Ant-App-DB application).

### Archived software files as at the time of publication

App-Ant-Database (DOI: 10.5281/zenodo.13223) ^40^; Dataset Ant-App-DB (DOI: 10.5281/zenodo.13225) ^41^; Dataplus Application (DOI: 10.5281/zenodo.13226) ^42^.

### License

All associated files are licensed under the Academic Free License 3.0 (AFL 3.0).

## Supplementary materials

Ant-App-DB: Work Flow, UML Designs, Configuration and Calculation Steps. Click here to access the file. http://dx.doi.org/10.5256/f1000research.5931.s45712
