# Supplementary material for: Ant-App-DB: a smart solution for monitoring arthropods activities, experimental data management and solar calculations without GPS in behavioral field studies
Source: F1000Res. 2015 May 12;3:311. Originally published 2014 Dec 19. [Version 3] doi: 10.12688/f1000research.5931.3 (PMC4416535; doi:10.12688/f1000research.5931.3)
Supplement: Peer review history

# Peer review: Referee response for version 2

**DOI:** 10.5256/f1000research.6814.r8339

**Published:** 2015-04-27

**Authors:** David White

**Affiliations:** 1 Department of Electrical and Computer Engineering, Clemson University, Clemson, SC, USA

The overall technical implementation of the software appears to be sound. I am not familiar with arthropod marking and tracking methods, thus the article could be improved by briefly outlining these methods in the introduction as to better align with the purpose/design of the Ant-App-DB. 

The paper does adequately explain methods and provides description of the underlying software framework through the use of figures and descriptions.

There are some issues that the authors need to address before indexing:

1. "dot Net framework" = .NET Framework
2. Figure 4 caption needs to be reviewed as it has several typos "Figure 2(b), Figure 2(c) etc..."
3. Figure 7 is difficult to read. It may be better to print on an entire page.
4. What is "(S-Figure 2li)" pg. 10
5. The discussion reads as a rough draft as there are several run-on sentences and typos. For example, "Several of Ant-App-DB's features compare favorably with other currently available solutions........ efficient insect monitoring such as ^2-8^." Is not a good style. Providing some examples of how these systems all compare would strengthen this discussion. see pg. 12.
6. The authors need to be careful with their subjective statements such as "Moreover, Ant-App-DB is more user friendly that (typo) other applications....." See critique #5. A more thorough review of existing systems is really needed if you are going to make these statements.  pg. 12. 

I have read this submission. I believe that I have an appropriate level of expertise to confirm that it is of an acceptable scientific standard.

# Response

**Published:** 2015-05-04

**Authors:** Zeeshan Ahmed

**Affiliations:** University of Massachusetts Medical School, USA

Authors > Thank you so much for your time in reviewing and approving our manuscript, and giving valuable suggestions, which all helped to further improve our manuscript.

Reviewer > The overall technical implementation of the software appears to be sound.

Authors > Thank you so much for appreciating our work.

Reviewer > I am not familiar with arthropod marking and tracking methods, thus the article could be improved by briefly outlining these methods in the introduction as to better align with the purpose/design of the Ant-App-DB.

Authors > Thanks for the nice suggestion and we agree with you. We have tried to revise it accordingly. The desert ants that we describe as an example for the use of the applications were individually marked with a three dot color code to be able to register individual ants at different locations and solar times. This information now is included at various positions in the text.

Reviewer > The paper does adequately explain methods and provides description of the underlying software framework through the use of figures and descriptions.

Authors > Thanks and we agree with you.

Reviewer > There are some issues that the authors need to address before indexing:

Authors > Thanks for pointing these issues and we have tried to clarify these in revision.

 

1. Reviewer > "dot Net framework" = .NET Framework

Authors > You are right, and we have corrected it.

1. Reviewer > Figure 4 caption needs to be reviewed as it has several typos "Figure 2(b), Figure 2(c) etc..."

Authors > You are right, and we have corrected it.

1. Reviewer > Figure 7 is difficult to read. It may be better to print on an entire page.

Authors > You are right, and we have provided new Figure with better resolution.

1. Reviewer > What is "(S-Figure 2li)" pg. 10

Authors > You are right, it’s a mistake and we have corrected it by “Figure 7i”.

1. Reviewer > The discussion reads as a rough draft as there are several run-on sentences and typos. For example, "Several of Ant-App-DB's features compare favorably with other currently available solutions........ efficient insect monitoring such as2-8." Is not a good style. Providing some examples of how these systems all compare would strengthen this discussion. see pg. 12.
2. Reviewer > The authors need to be careful with their subjective statements such as "Moreover, Ant-App-DB is more user friendly that (typo) other applications....." See critique #5. A more thorough review of existing systems is really needed if you are going to make these statements.  pg. 12.

 Authors > You are right and we have revised it.

# Peer review: Referee response for version 2

**DOI:** 10.5256/f1000research.6814.r8452

**Published:** 2015-04-24

**Authors:** Harald Wolf

**Affiliations:** 1 Institute of Neurobiology, University of Ulm, Ulm, Germany

This manuscript presents an interesting and potentially powerful new tool for monitoring behavioral data on small animals, particularly arthropods in field experiments. This includes remote areas without internet or situations without GPS access. The basic idea and the present implementation appear useful indeed for field studies on animal navigation and related topics. It is a major advantage that the modular structure of the application should allow expansion towards related applications and the implementation of additional features, depending on the particular user requirements.

There are, however, a few shortcoming, most of them minor, but also a couple of more serious ones. I shall list them below, roughly arranged in the sequence of their importance:

 

- Data input: While the text explains in some detail the modular structure of Ant-App-DB and the basic function of the different modules, it remains mostly unclear to me what kind of input data the application would accept. Perhaps the most important features when studying arthropod activity are track recordings, or the monitoring of locomotor paths. Can such data be recorded with Ant-App-DB? Or is it just single coordinates in space and time? Or is it simply numbers that represent something the researcher may be interested in, such as walking distance, walking times etc.? Considerable effort is spent to explain, for example, the solar estimations. This is good, particularly for the uninitiated reader and naturally for research topics where solar azimuth and similar data are important. It would be highly desirable to spend similar effort on the explanation of data recording itself and the different options that exist and possible simple expansions that may be possible.
- Parts of the text are written in good English, quite crisp and altogether clear. Other parts of the text, by contrast, suffer from a dense style with long-winding sentences and some unintelligible sentence structure. Together with the occasional lab jargon or computer speak, this makes parts of the text somewhat difficult to read and understand.
- The English language is not always correct, despite native English speaker correction at some point in manuscript evolution. Hyphenation is just one example here. Typographic errors may also fall into this category; e.g. figure numbers in legend of fig.4 are consistently wrong.

I have read this submission. I believe that I have an appropriate level of expertise to confirm that it is of an acceptable scientific standard, however I have significant reservations, as outlined above.

# Response

**Published:** 2015-05-04

**Authors:** Zeeshan Ahmed

**Affiliations:** University of Massachusetts Medical School, USA

Authors > Thank you so much for your time in reviewing our manuscript and giving valuable suggestions, which all helped to further improve our manuscript.

Reviewer > This manuscript presents an interesting and potentially powerful new tool for monitoring behavioral data on small animals, particularly arthropods in field experiments. This includes remote areas without internet or situations without GPS access. The basic idea and the present implementation appear useful indeed for field studies on animal navigation and related topics. It is a major advantage that the modular structure of the application should allow expansion towards related applications and the implementation of additional features, depending on the particular user requirements.

Authors > Thank you so much for appreciating our work and we agree with you.

Reviewer > There are, however, a few shortcoming, most of them minor, but also a couple of more serious ones. I shall list them below, roughly arranged in the sequence of their importance:

Authors > Thank you so much for raising minor and major points.

 

- Reviewer > Data input: While the text explains in some detail the modular structure of Ant-App-DB and the basic function of the different modules, it remains mostly unclear to me what kind of input data the application would accept. Perhaps the most important features when studying arthropod activity are track recordings, or the monitoring of locomotor paths.

Authors > Our application helps in recording the information about individually color-marked ants (three dot color code), their visits to the feeders and the respective solar estimations. It does not help in the monitoring of locomotor paths. However, it can also be used with the placement of multiple feeders, for example to track at which locations the ant was.

Reviewer > Can such data be recorded with Ant-App-DB? Or is it just single coordinates in space and time? Or is it simply numbers that represent something the researcher may be interested in, such as walking distance, walking times etc.?

Authors > You are right, it records only coordinates and numbers of individually registered ants, but we are keeping this as a very valuable suggestion by you for our future work. Due to the time and resource constrains, we cannot implement sophisticated locomotion path tracking features now. This certainly is an interesting aspect for future developments as we point out in the discussion.   

Reviewer > Considerable effort is spent to explain, for example, the solar estimations. This is good, particularly for the uninitiated reader and naturally for research topics where solar azimuth and similar data are important.

Authors > Thanks and we agree with you.

Reviewer > It would be highly desirable to spend similar effort on the explanation of data recording itself and the different options that exist and possible simple expansions that may be possible.

Authors > We totally agree with you. As the scope of this paper was limited to the software/tool presentation (category: Software Tool Article), we did not provide detailed explanations of biological experimental procedures. We appreciate your suggestion and certainly will implement this in future reports on biological applications.

 

- Reviewer > Parts of the text are written in good English, quite crisp and altogether clear. Other parts of the text, by contrast, suffer from a dense style with long-winding sentences and some unintelligible sentence structure. Together with the occasional lab jargon or computer speak, this makes parts of the text somewhat difficult to read and understand.

Authors > Thanks for the nice suggestion and we agree with you. We have revised and tried to improve the content presentation.

 

- Reviewer > The English language is not always correct, despite native English speaker correction at some point in manuscript evolution. Hyphenation is just one example here. Typographic errors may also fall into this category; e.g. figure numbers in legend of fig.4 are consistently wrong.

 

Authors > Thanks for the suggestion and we agree with you. We have revised and improved the content presentation as well as corrected Figure 4 legend.

# Peer review: Referee response for version 2

**DOI:** 10.5256/f1000research.6814.r8266

**Published:** 2015-04-20

**Authors:** Charles Bargeron

**Affiliations:** 1 Agriculture and Natural Resources, University of Georgia, Tifton, GA, USA

Article was very good and provided the needed technical and biological information to describe the problem and solutions. However, it was unclear to me why GPS could not be used, since it does not require the need for cellular service with most devices. Also why was the data not stored on the smartphone and then uploaded to a web server when internet access was available? This would allow for data from multiple users to be combined and could be outputted to Excel without the creation of another desktop application. There may be good reasons for why both of these solutions were not considered but I didn't feel they were addressed as part of the article.

I have read this submission. I believe that I have an appropriate level of expertise to confirm that it is of an acceptable scientific standard, however I have significant reservations, as outlined above.

# Response

**Published:** 2015-05-04

**Authors:** Zeeshan Ahmed

**Affiliations:** University of Massachusetts Medical School, USA

Authors > Thank you so much for your time in reviewing our manuscript and giving valuable suggestions, which all helped to further improve our manuscript.

Reviewer > Article was very good and provided the needed technical and biological information to describe the problem and solutions.

Authors > Thank you so much for appreciating our work and we agree with you.

Reviewer > However, it was unclear to me why GPS could not be used, since it does not require the need for cellular service with most devices.

Authors > You are absolutely right in your point. In general, the known GPS coordinates of the location where the insects are studied can be used as an input to the system. However, our point was that this application can be used without online GPS service. We have tried to contribute for such cases, where scientists/observers/users do not have a GPS facility with them due to any reason, e.g. if it is costly, not compatible to the in use devices, not providing the spatial resolution needed, etc. Following your suggestion, we have highlighted this in our revision.

Reviewer > Also why was the data not stored on the smartphone and then uploaded to a web server when internet access was available? This would allow for data from multiple users to be combined and could be outputted to Excel without the creation of another desktop application. There may be good reasons for why both of these solutions were not considered but I didn't feel they were addressed as part of the article.

Authors > You are absolutely right and in fact it is possible. Data is stored inside the Smartphone memory and, potentially, can be shared via internet access, USB connection, Bluetooth etc. Moreover, it is also possible to combine multiple data files. We have not implemented direct data sharing options in our smart phone application because our data, which is stored in the internal or external memory card, can easily be transferred or shared using existing Android smart phone features. Following your suggestion, we have highlighted this in our revision.

Authors > Thank you so much for your suggestions, we are keeping these for our future development tasks.
